# Supplementary material for: Detailed molecular and epigenetic characterization of the pig IPEC-J2 and chicken SL-29 cell lines
Source: iScience. 2023 Feb 20;26(3):106252. doi: 10.1016/j.isci.2023.106252 (PMC10018572; doi:10.1016/j.isci.2023.106252)
Supplement: Data S1. Complete homer output for identified motifs in Pig IPECJ-2, related to Table 2 — Homer motif analysis results for histone modifications H3K4me1, H3K4me3, H3K27ac, and enhancer elements of pig IPECJ2 cell line. P-values >1e-10 are possible false positives. Within each folder (e.g. peak_files_CTCF) are the html files showing the identified motifs when using homer (e.g. homerResults.html). [file mmc2.zip › S5/Pig_IPECJ_2/peak_files_H3K27ac/homerResults/motif4.similar.html]

motif4

## Information for motif4

A
G
T
C
G
T
A
C
A
C
T
G
A
T
C
G
C
G
A
T
C
G
T
A
T
G
C
A
C
A
T
G
  
Reverse Opposite:  

G
T
A
C
A
C
G
T
C
G
A
T
C
G
T
A
A
T
G
C
A
G
T
C
A
C
T
G
T
C
A
G
  

|  |  |
| --- | --- |
| p-value: | 1e-110 |
| log p-value: | -2.543e+02 |
| Information Content per bp: | 1.758 |
| Number of Target Sequences with motif | 12377.0 |
| Percentage of Target Sequences with motif | 23.54% |
| Number of Background Sequences with motif | 10150.6 |
| Percentage of Background Sequences with motif | 19.57% |
| Average Position of motif in Targets | 149.2 +/- 84.7bp |
| Average Position of motif in Background | 148.0 +/- 91.4bp |
| Strand Bias (log2 ratio + to - strand density) | 0.0 |
| Multiplicity (# of sites on avg that occur together) | 1.13 |
| Motif File: | file (matrix) reverse opposite |

### Similar de novo motifs found

|  |  |  |  |  |  |  |  |
| --- | --- | --- | --- | --- | --- | --- | --- |
| Rank | Match Score | Redundant Motif | P-value | log P-value | % of Targets | % of Background | Motif file |
| 1 | 0.612 | T A G C A T C G C T A G A T G C C T A G C A T G G C A T G C T A | 1e-58 | -135.159224 | 18.21% | 15.59% | motif file (matrix) |
| 2 | 0.764 | A T G C A T G C G A T C C T A G T A C G C A G T C T G A G T A C C A T G G A C T | 1e-57 | -132.334385 | 15.72% | 13.28% | motif file (matrix) |
| 3 | 0.710 | A T G C G A C T T A G C C G T A C T A G T C A G G A C T C T G A T C G A A G C T | 1e-46 | -107.080315 | 14.20% | 12.10% | motif file (matrix) |
